# Supplementary material for: Combination of Bone Marrow Biopsy and Flow Cytometric Analysis: The Prognostically Relevant Central Approach for Detecting Bone Marrow Invasion in Diffuse Large B-Cell Lymphoma
Source: Diagnostics (Basel). 2021 Sep 20;11(9):1724. doi: 10.3390/diagnostics11091724 (PMC8470419; doi:10.3390/diagnostics11091724)
Supplement: Supplementary file 1 [file diagnostics-11-01724-s001.zip › diagnostics-1381920-supplementary.pdf]

Supplementary Table S1. Impact of the detection of BM involvement by single or combinatory modality on overall survival

		Univariate analysis			Multivariate analysis*			
**Modality**	**Result (n)**	**HR**	**95% CI**	**p**	**HR**	**95% CI**	**p**	**c-index**
BM biopsy	− (170)	1	–	–	1	–	–	
	+ (14)	5.22	2.61–10.47	<0.001	3.00	1.31–6.84	0.009	0.738
BM clot	− (172)	1	–	–	1	–	–	
	+ (12)	3.44	1.67–7.07	<0.001	2.03	0.92–4.47	0.079	0.735
FCM	− (166)	1	–	–	1	–	–	
	+ (18)	2.24	1.13–4.45	0.021	1.86	0.92–3.74	0.082	0.74
BM smear	− (158)	1	–	–	1	–	–	
	+ (26)	2.16	1.18–3.97	0.0013	1.27	0.65–2.46	0.483	0.729
BM biopsy and/or clot	− (186)	1	–	–	1	–	–	
	+ (16)	3.78	1.94–7.35	<0.001	2.48	1.18–5.21	0.017	0.745
BM biopsy and/or FCM	− (156)	1	–	–	1	–	–	
	+ (28)	3.26	1.84–5.77	<0.001	2.37	1.28–4.40	0.006	0.748
BM biopsy and/or smear	− (152)	1	–	–	1	–	–	
	+ (32)	2.43	1.38–4.31	0.002	1.51	0.81–2.81	0.197	0.734
BM clot and/or FCM	− (158)	1	–	–	1	–	–	
	+ (26)	2.72	1.53–4.85	<0.001	1.95	1.06–3.59	0.033	0.742
BM clot and/or smear	− (154)	1	–	–	1	–	–	
	+ (30)	2.48	1.40–4.39	0.002	1.56	0.83–2.91	0.164	0.738
FCM and/or BM smear	− (146)	1	–	–	1	–	–	
	+ (38)	2.27	1.32–3.90	0.003	1.60	0.89–2.86	0.114	0.741
BM biopsy, clot, and/or FCM	− (155)	1	–	–	1	–	–	
	+ (29)	2.77	1.57–4.88	<0.001	2.03	1.11–3.72	0.021	0.746
BM biopsy, clot, and/or smear	− (151)	1	–	–	1	–	–	
	+ (33)	2.56	1.46–4.48	0.001	1.65	0.89–3.04	0.112	0.742
BM biopsy, FCM, and/or smear	− (142)	1	–	–	1	–	–	
	+ (42)	2.45	1.44–4.16	<0.001	1.71	0.97–3.04	0.066	0.742
BM clot, FCM, and/or smear	− (144)	1	–	–	1	–	–	
	+ (40)	2.55	1.50–4.33	<0.001	1.77	1.00–3.14	0.05	0.744
BM biopsy, clot, FCM, and/or smear	− (142)	1	–	–	1	–	–	
	+ (42)	2.45	1.44–4.16	<0.001	1.71	0.97–3.04	0.066	0.742

BM, bone marrow; CI, confidence interval; HR, hazard ratio; −, negative; +, positive; * adjusting for IPI factors.

Supplementary Table S2. Cox proportional-hazards regression analysis for PFS

		**Univariate analysis**			**Multivariate analysis ***			
**Modality**	Result (n)	HR	95% CI	p	HR	95% CI	p	c-index
**PET-CT**	Negative (124)	1	–	–	1	–	–	
	Positive (30)	3.03	1.76–5.22	<0.001	1.77	0.92–3.43	0.089	0.680

PET-CT, positron emission tomography of 18F-fluorodeoxyglucose and computed tomography; PFS, progression-free survival *adjusted by IPI factors (age, LDH, PS 2–4, disease stage III and IV, and extranodal lesion).
